# Long-term treatment outcomes of intermittent androgen deprivation therapy for relapsed prostate cancer after radical prostatectomy

**DOI:** 10.1371/journal.pone.0197252

**Published:** 2018-05-24

**Authors:** Shintaro Maru, Hideki Uchino, Takahiro Osawa, Satoshi Chiba, Gaku Mouri, Ataru Sazawa

**Affiliations:** 1 Department of Urology, Obihiro-Kosei General Hospital, Obihiro, Hokkaido, Japan; 2 Department of Urology, Jinyukai Hospital, Sapporo, Hokkaido, Japan; 3 Department of Renal and Genitourinary Surgery Graduate School of Medicine, Hokkaido University Sapporo, Hokkaido, Japan; National Health Research Institutes, TAIWAN

## Abstract

**Purpose:**

Intermittent androgen deprivation therapy is an effective treatment for metastatic prostate cancer. However, no study to date has evaluated the long-term outcomes of this treatment among patients with prostate cancer after radical prostatectomy. We retrospectively examined the treatment outcomes of patients with prostate-specific antigen recurrence who underwent radical prostatectomy at our department.

**Materials and methods:**

Of the 690 patients who underwent radical prostatectomy for local prostate cancer between 1988 and 2011, 129 patients who received androgen deprivation therapy for prostate-specific antigen recurrence were included in this study. Patient characteristics, luteinizing hormone-releasing hormone agonist administration, and outcomes were compared between the intermittent androgen deprivation group (n = 66) and the continuous androgen deprivation therapy group (n = 63). The non-recurrence and overall survival rates were compared between groups.

**Results:**

Thirty-six patients (27.9%) experienced recurrence after luteinizing hormone-releasing hormone agonist administration. The 5-year non-recurrence rate and 10-year overall survival rate were higher in the intermittent group (92.9%) than in the continuous group (92.9 vs 57.9%, P < 0.001; and 95.9% vs 84.3%, P = 0.047, respectively). Furthermore, 63 patients (48.8%) showed a PSA nadir of less than 0.01 ng/mL after initiation of luteinizing hormone-releasing hormone agonist; among these patients, the non-recurrence rate was significantly higher in the intermittent androgen deprivation group (P = 0.003).

**Conclusions:**

Intermittent androgen deprivation therapy for prostate specific antigen recurrence after radical prostatectomy contributed to improvement of the non-recurrence rate and overall survival, and can be considered an effective therapy for better prognosis.

## Introduction

In the 1940s, Huggins et al demonstrated for the first time that androgen suppression via bilateral orchiectomy relieved the symptoms of prostate cancer (PCa) [1]. Since then, the standard treatment for metastatic PCa has been androgen deprivation therapy (ADT). It has been reported that biochemical effects are achieved in at least 90% of patients who undergo ADT [2] and that clinical effects are achieved in 70–80% of patients [3]. However, there are some reports that such effects are temporary and that at least 50% of patients experience recurrence within two years [4,5]. In addition, because of the necessity of continuous treatment, ADT increases the risk of various side effects including hot flashes, fatigue, depression, erectile dysfunction, debility sexualis, and gynecomastia [6].

In response to the limitations described above, intermittent androgen deprivation therapy (IAD) became an alternative to continuous androgen deprivation therapy (CAD) in the 1990s [7,8]. There are many reports that IAD for metastatic PCa is effective at minimizing side effects and reducing medical cost [9,10,11]. In the recent guidelines by the European Association of Urology, IAD is recommended as the first line therapy. However, it has also been reported that outcomes and prognosis of IAD are not necessarily equivalent to those of CAD [12]. In contrast, there are few reports on the therapeutic effect and prognosis of IAD for PCa after radical prostatectomy. Thus, examination of long-term results of IAD for PSA recurrence after radical prostatectomy for PCa is not common clinical practice. In this study, we examined the treatment outcomes of IAD with those of luteinizing hormone-releasing hormone agonist (LHRHa) alone, which was administered to patients with PSA recurrence after radical prostatectomy at our department, and retrospectively compared these results with the treatment outcomes of patients receiving CAD.

## Materials and methods

Of 690 patients who underwent radical prostatectomy for PCa between 1988 and 2011, 129 patients who received androgen deprivation therapy for PSA recurrence were included in this study. At the Department of Urology Obihiro-kosei Hospital, Japan, the following procedures are followed for IAD therapy: LHRHa alone is administered to patients who show increased PSA level after radical prostatectomy; LHRHa is discontinued when the PSA level is maintained near the level of sensitivity of the PSA test; and LHRHa is resumed when the PSA level increases again. Patients were treated with either of the following LHRH analogues: leuprolide acetate or goserelin acetate. In this study, recurrence was defined as requirement of an additional anti-androgenic agent after the initiation of LHRHa. One cycle of IAD was defined as the time on treatment and time off treatment. This study was approved by the Committee on the Ethics of Obihiro-kousei General Hospital (Permit Number: 2016–067). All data were fully anonymized before we accessed them and the ethics committee waived the requirement for informed consent.

Patients were divided into two groups: the IAD group (66 patients, 51.2%) and CAD group (63 patients, 48.8%). Patient characteristics, observation periods, actual LHRHa administration, and outcomes were compared between the two groups using the Mann Whitney U test and Student t test. In addition, in the IAD group (66 patients), we examined the frequency of LHRHa administration, time on treatment, time off treatment, and the change in PSA level between the initiation and completion of LHRHa. Differences in five-year non-recurrence rate and 10-year overall survival rate were compared between the IAD group and the CAD group. Patients were classified based on Gleason Score (GS) 7 or less and GS 8 or more, and the non-recurrence and overall survival rates were compared between the two classifications. In the patients who showed a PSA nadir of < 0.01 ng/mL after the initiation of LHRHa, the non-recurrence rate was compared between the two groups.

The non-recurrence rate and the survival rate between the two groups were examined using the Kaplan-Meier method and log-rank test. Statistical Analyses were performed using Stat Mate IV and all P-value less than 0.05 was considered statistically significant.

## Results

Table 1 shows the patient characteristics of the two groups. No significant difference was found in the median age of the two groups (67 years in the IAD group; 68 years in the CAD group). There was also no significant difference in median preoperative PSA level (13.5 ng/mL in the IAD group; 11.8 ng/mL in the CAD group). The median observation period between the initiation of LHRHa and the completion of follow-up was 75 and 56 months in the IAD and CAD groups, respectively. The period after radical prostatectomy until completion of follow-up was 127 and 91 months in the IAD and CAD groups, respectively. All of these periods were significantly longer in the IAD group. Concerning pathological findings, the GS was significantly lower in the IAD group (52 patients with GS ≤ 7 in the IAD group; 27 patients with GS ≤ 7 in the CAD group). On the other hand, the number of the patients with GS ≥ 8 was significantly higher in the CAD group (36 patients) than in the IAD group (14 patients). There was no significant difference in the T classification and the presence or absence of surgical margin-positive tumors between groups.

**Table 1:** Overall characteristics of all patients.

Characteristic	IAD (n = 66)	CAD (n = 63)	P Value
Median Age (yr)	67	68	N.S
Prior operation median PSA level (ng/ml)	13.5	11.8	N.S
Prior hormone therapy (%)	21.2	11.1	N.S
Median Follow up (mo)	127	91	<0.001
Median ADT follow up (mo)	75	56	0.019
Gleason Score (n)			
6≦	22	9	0.011
7	30	18	0.047
8≧	14	36	<0.001
T Stage (n)			
2	21	25	N.S
3	43	37	N.S
Unknown	2	1	N.S
Surgical margin (n)			
Positive	30	39	N.S
Negative	34	23	N.S
Unknown	2	1	N.S

Table 2 shows the actual LHRHa administration and outcomes between the two groups. The median time between radical prostatectomy and the initiation of LHRHa was significantly longer in the IAD group (48 months) than in the CAD group (23 months). There was no significant difference in the median PSA level at initial LHRHa administration between the IAD group (2.02 ng/mL) and CAD group (2.10 ng/mL). For actual LHRHa administration, the median duration after the initiation of first LHRHa until PSA nadir was significantly shorter in the IAD group (5 months) than in the CAD group (9 months). The median PSA nadir was significantly lower in the IAD group. The number of the patients who showed a PSA nadir of less than 0.01 ng/mL after initial LHRHa administration was significantly higher in the IAD group (60.6%) than in the CAD group (36.5%). Concerning outcomes, the number of the patients who experienced recurrence and required the administration of an anti-androgenic agent was significantly lower in the IAD group (8 patients, 12.1%) than in the CAD group (28 patients, 44.4%). The number of cancer-related deaths was significantly lower in the IAD group (2 patients, 3.0%) than in the CAD group (8 patients, 12.7%). However, the number of deaths due to other causes was higher in the IAD group.

**Table 2:** Characteristics of patients treated with IAD and CAD.

Characteristic	IAD (n = 66)	CAD (n = 63)	P Value
Prior ADT			
Median PSA level (ng/ml)	2.02	2.10	0.07
Interval between RP and ADT (mo)	48	23	<0.001
			
Posterior ADT			
Median PSA nadir level	0.01	0.03	0.003
Interval between ADT and PSA nadir (mo)	5	9	<0.001
Reached PSA≦0.01 (%)	60.0	36.5	0.006
			
Outcome			
Recurrence (n)	8	28	<0.001
Cancer death (n)	2	8	0.013

**Fig 1:**
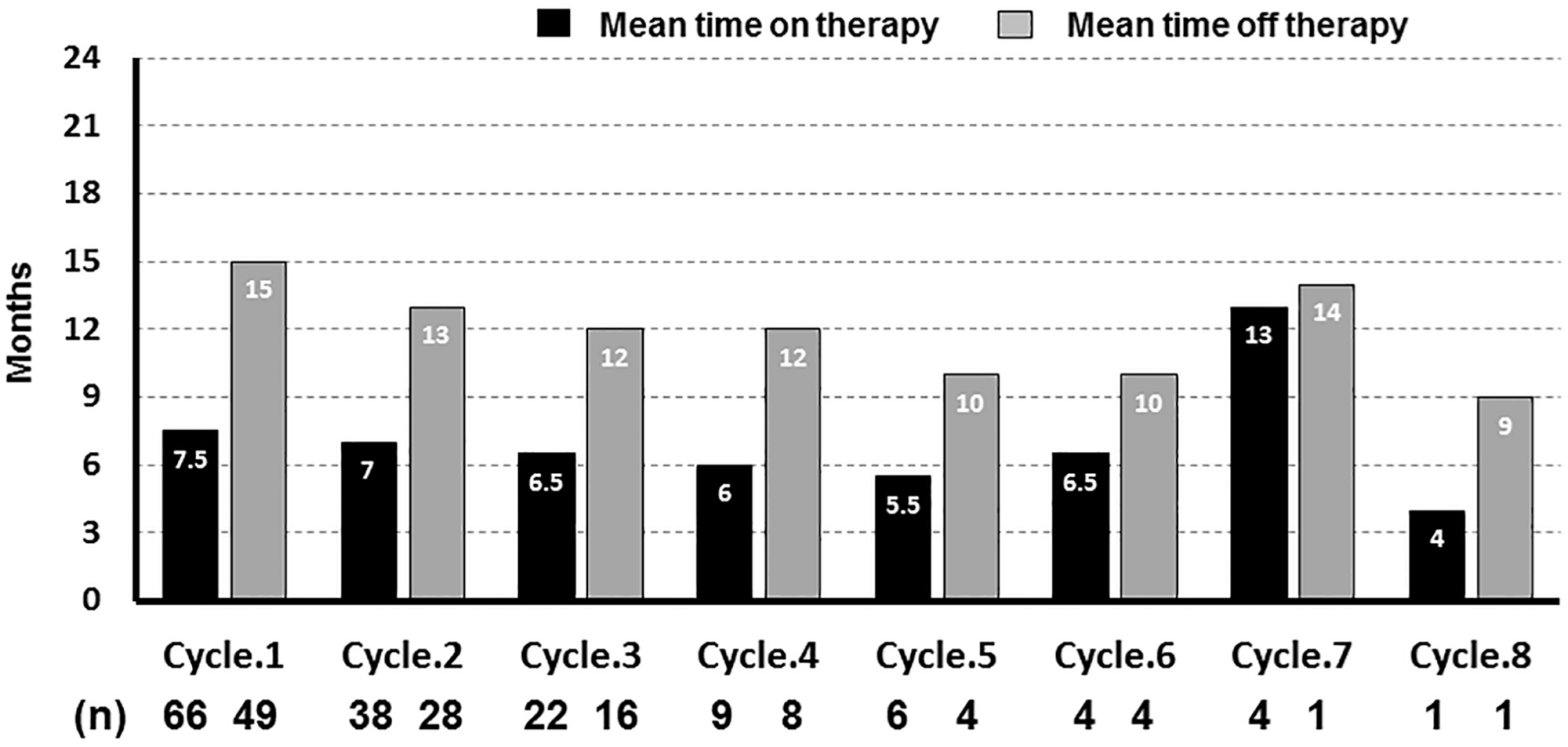
The median duration of time on treatment or time off treatment in the IAD group.

**Fig 2:**
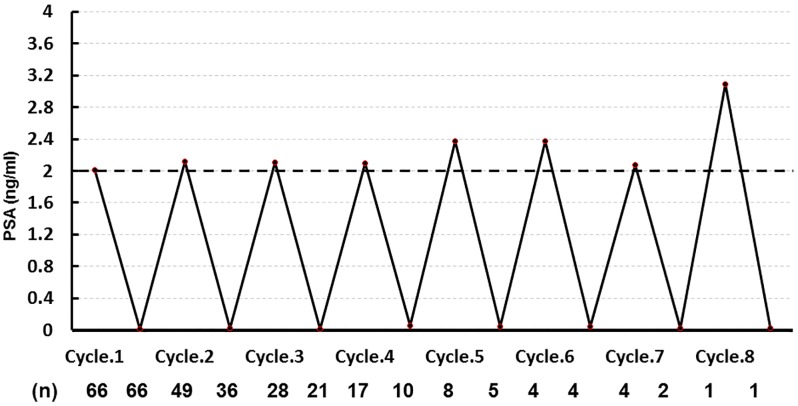
Mean PSA level prior to posterior LHRHa therapy in the IAD group.

In the IAD group, the median frequency of LHRHa administration was two times (range 1–8 times); the median duration of administration was 6.5 months (range 3–64 months); and the median duration of drug holiday was 12 months (range 2–72 months) (Fig 1). Concerning change in the median PSA level per cycle, the median level at the initiation or resumption of LHRHa in all 8 cycles was 2.12 ng/mL (range 2.02–3.09 ng/mL), while the median level at completion of LHRHa was 0.02 ng/mL (range 0.01–0.06 ng/mL) (Fig 2).

**Fig 3:**
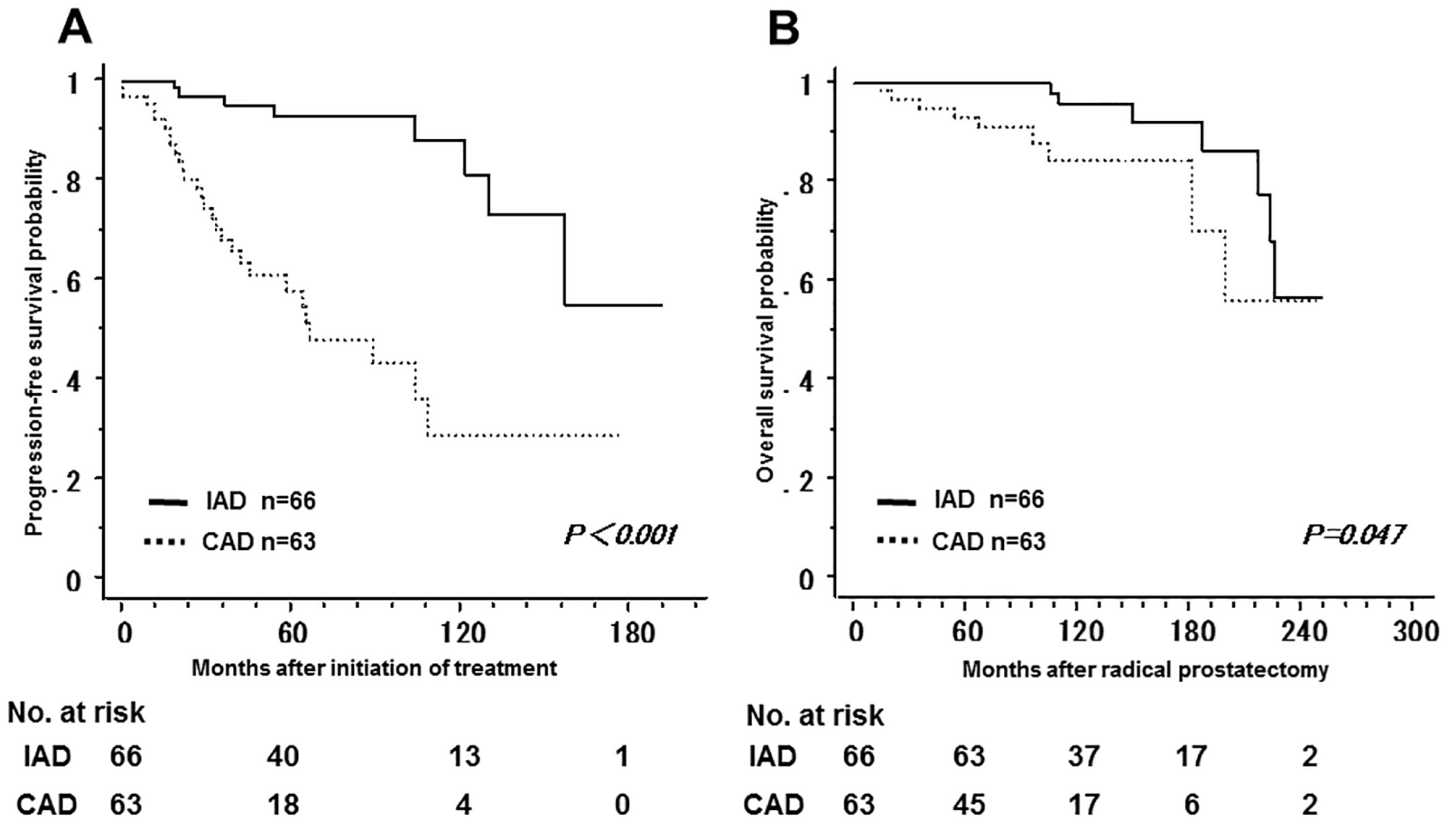
The non-recurrence rate and survival rate of the IAD group and the CAD group. Kaplan-Meier analysis of progression-free survival (A) and overall survival (B) in the IAD group and CAD group.

**Fig 4:**
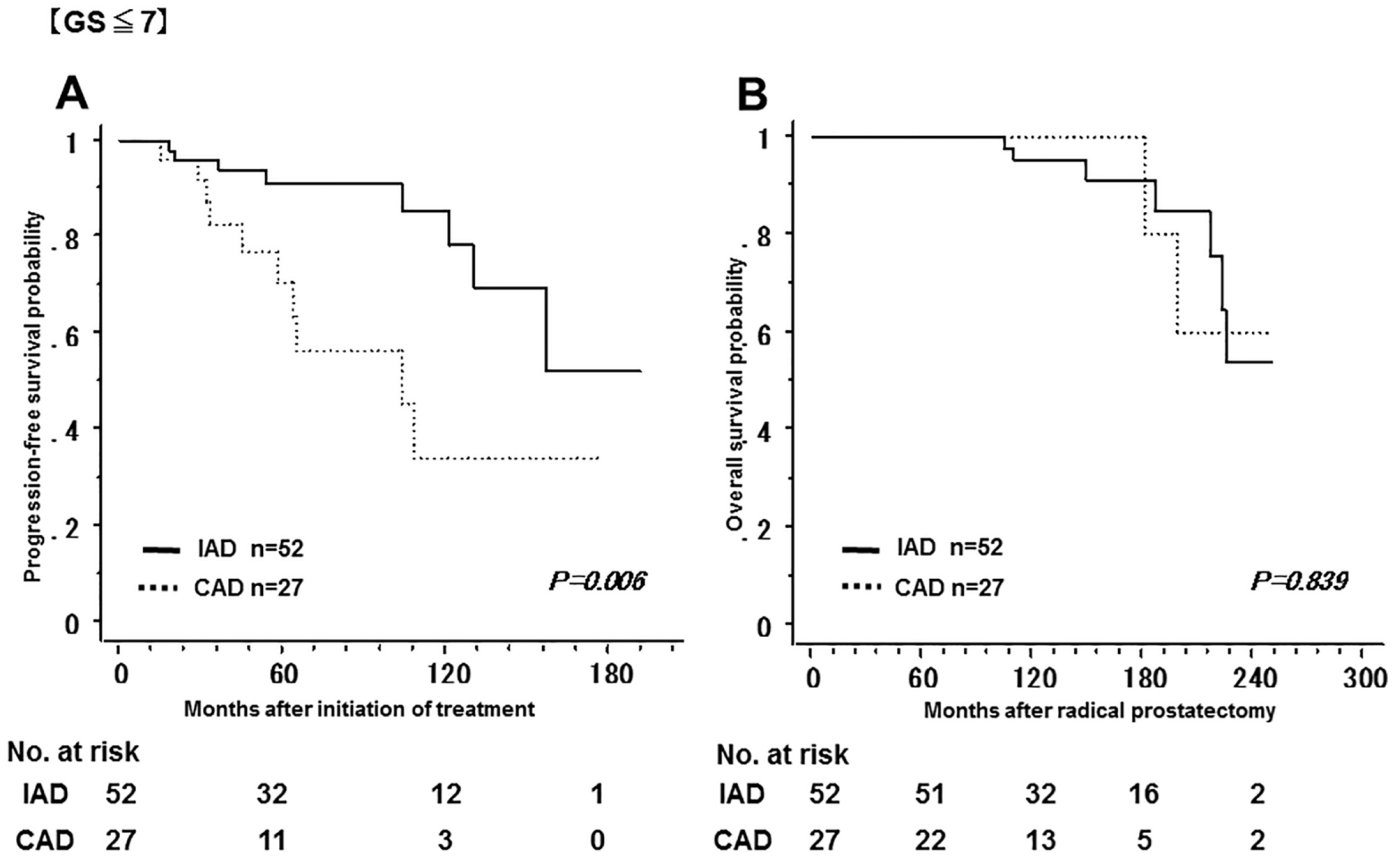
The non-recurrence rate and survival rate of the IAD group and the CAD group with Gleason Score ≤ 7. Kaplan-Meier analysis of progression-free survival (A) and overall survival (B) in both two groups in patients with Gleason Score ≤ 7.

**Fig 5:**
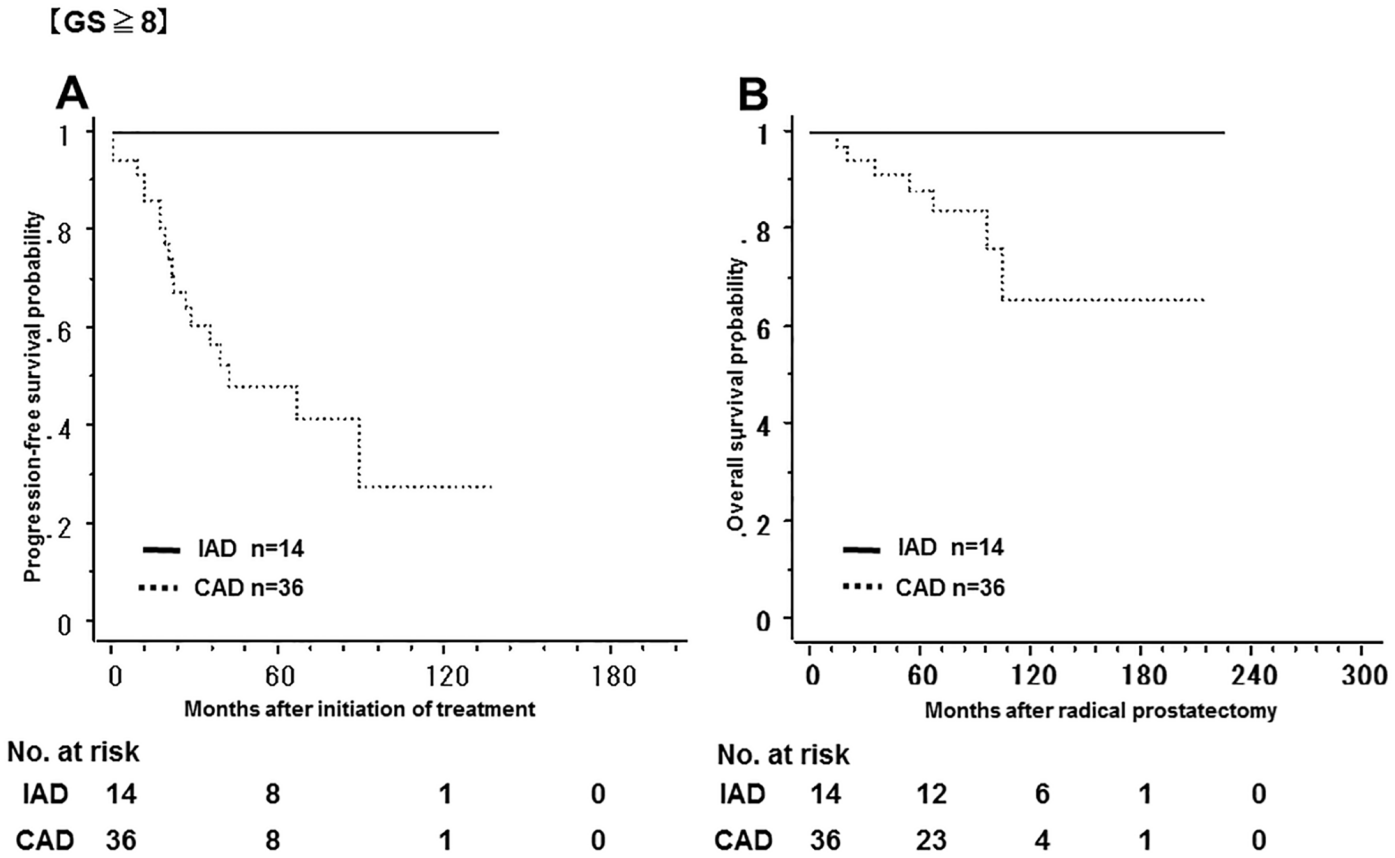
The non-recurrence rate and survival rate of the IAD group and the CAD group with Gleason Score ≥ 8. Kaplan-Meier analysis of progression-free survival (A) and overall survival (B) in both two groups in patients with Gleason Score ≥ 8.

**Fig 6:**
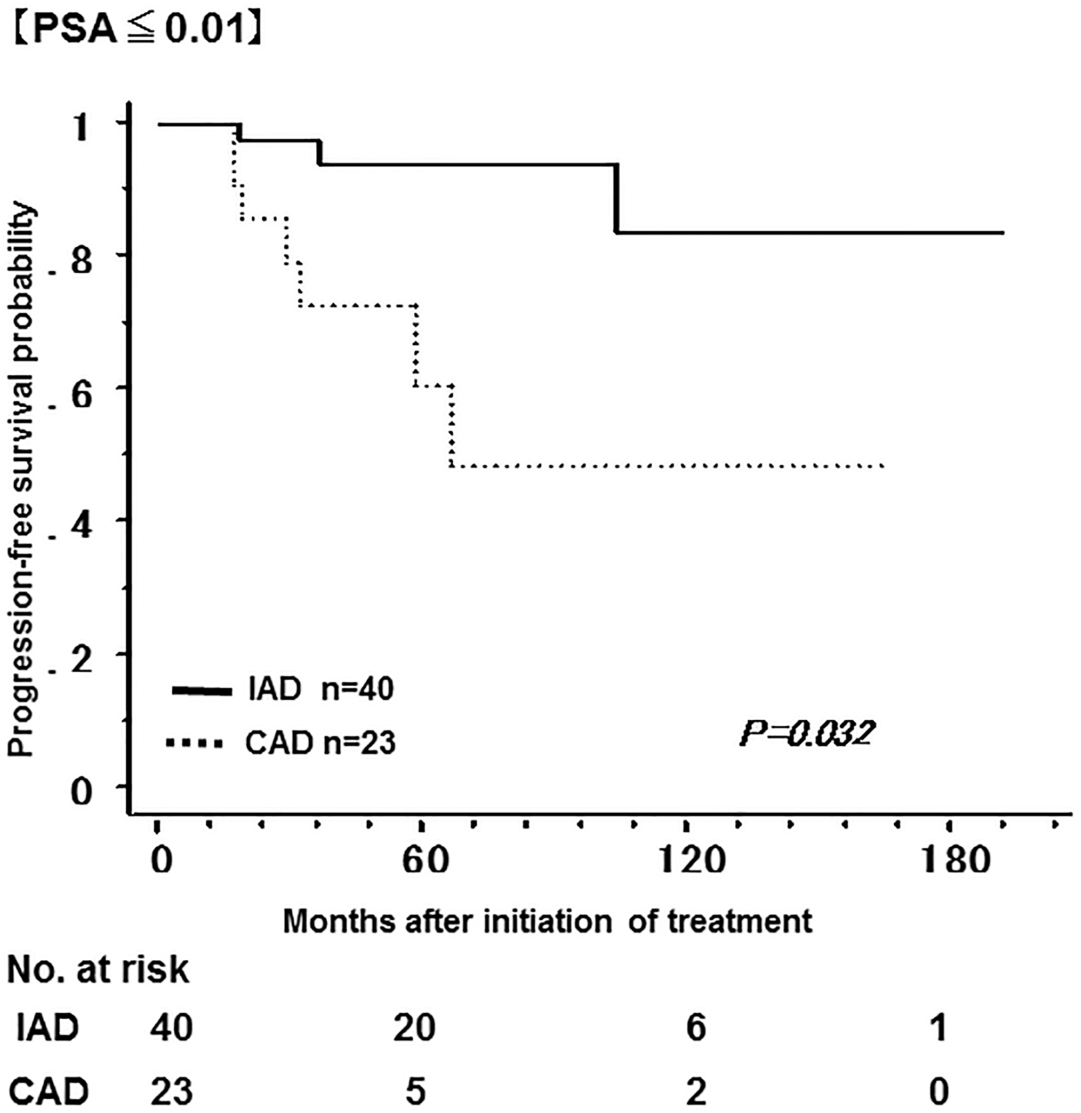
The non-recurrence rate of the IAD group and the CAD group with PSA ≤ 0.01 ng/ml. Kaplan-Meier analysis of progression-free survival in the patients who showed a PSA nadir of less than 0.01 ng/ml after LHRHa administration.

Fig 3 shows the non-recurrence rate and survival rate of the IAD group and the CAD group. The 5-year non-recurrence rate was significantly higher in the IAD group (92.9%) than in the CAD group (57.9%). In addition, the 10-year overall survival rate was significantly higher in the IAD group (95.9%) than in the CAD group (84.3%). Tables 1 and 2 show a bias in the pathologic characteristics and in the number of the patients with a PSA nadir of less than 0.01 ng/mL after LHRHa administration between the two groups; the number of the patients with GS ≤ 7 was higher in the IAD group, while the number of the patients with GS ≥ 8 was higher in the CAD group. Therefore, the patients were classified into GS ≤ 7 and GS ≥ 8 in order to compare the non-recurrence rate and the overall survival rate between the two groups. Among patients with GS ≤ 7, the 5-year non-recurrence rate was 91.2% and 70.7% in the IAD and CAD groups, respectively; the non-recurrence rate was significantly lower in the IAD group (Fig 4). In contrast, there was no significant difference in overall survival between groups. In addition, among patients with GS ≥ 8, both the 5-year non-recurrence rate and the 10-year overall survival rate was better in the IAD than in the CAD group (Fig 5). The 5-year non-recurrence rate was significantly higher in the IAD group (94.0%) than in the CAD group (48.4%) only among patients who showed a PSA nadir of < 0.01 ng/mL after LHRHa administration (Fig 6).

## Discussion

In this study evaluating the effectiveness of IAD therapy for relapsed PCa after radical prostatectomy, IAD therapy showed better overall survival compared to CAD therapy. Currently, there are many reports on the efficacy of IAD therapy for metastatic PCa. According to a review of the data obtained from 4668 subjects in eight randomized control studies, there was no significant difference in survival outcomes between IAD and CAD [13]. In a review of the data obtained from 5508 subjects in nine studies, overall survival and progression-free survival for IAD compared favorably with that of CAD. It was estimated that the median medical cost could be decreased by approximately 48% with IAD [11]. In contrast, according to a large-scale prospective study by Hussain et al, the median survival time after completion of 10 years of treatment in 1,535 patients with metastatic PCa was 5.8 and 5.1 years in the CAD and IAD groups, respectively (hazard rate [HR] 1.10; 90% confidence interval 0.99–1.23), indicating a 10% greater risk of death in the IAD group [12]. Therefore, at present, there is no definitive protocol established for IAD therapy.

On the other hand, there are few reports on the use of IAD for local PCa. Crook et al described the outcomes of patients who underwent IAD for PSA recurrence after PCa radiotherapy; the authors reported a median overall survival of 8.8 and 9.1 years in the IAD and CAD groups, respectively, with no significant difference between the two groups (HR 1.02; 95% confidence interval 0.86–1.21) [10]. In a prospective study performed in patients with PSA recurrence after radical prostatectomy, the authors did not provide data on cancer-specific survival and overall survival, but they reported no significant difference in androgen-independent progression between the IAD and CAD groups [14]. To our knowledge, ours is the first study to demonstrate the utility of IAD based on long-term follow-up of patients who underwent radical prostatectomy for PCa from the viewpoint of survival outcomes.

As shown in Fig 2, when analysis was limited to patients in the IAD group, the median PSA level at initiation or resumption of LHRHa in all eight cycles was 2.12 ng/mL, while the median PSA level at completion of LHRHa was 0.02 ng/mL. Based on various reports, the common timing for initiation or resumption of LHRHa therapy in PCa patients with distant metastasis was defined variably as follows: PSA level of at least 10 ng/mL; PSA level of at least 10 ng/mL with symptoms; or PSA level of at least 20 ng/mL. In addition, completion of LHRHa was defined as PSA level of 4.0 ng/mL or less [10,12,15,16,17]. In contrast, Tunn et al reported LHRHa administration was resumed when a patient showed PSA recurrence and PSA level was 3.0 ng/mL after radical prostatectomy; and LHRHa was discontinued when the PSA level was 0.5 ng/mL or less [14]. As mentioned previously, there was no significant difference in androgen-independent progression between the IAD and CAD groups. In the studies where LHRHa was resumed when the PSA level was 2.0 ng/mL, as in the current study, when the timing of resumption of LHRHa for patients with distant metastasis and PSA failure after radical prostatectomy was defined as PSA 2.0 ng/mL, the number of patients without distant metastasis in the CAD group (85%) was equivalent to that in the IAD group (85%). In patients with distant metastasis, 5-year progression free survival was 75% and 20% in the CAD and IAD groups, respectively [18]. Based on these data, resumption of treatment at a PSA level of 2.0 ng/mL is thought to be desirable for patients without distant metastasis.

With respect to duration of administration in our study, the median duration of time on treatment was 6.5 months and median duration of time off treatment was 12 months in the IAD group (Fig 1). Laitinen et al reported that no apoptosis was observed in the first three months, though cell proliferation did not persist 6–8 months after the initial treatment with IAD for patients with PCa [19]. Consequently, based on these results, the duration of LHRHa administration in our study was acceptable; the resumption of LHRHa due to PSA failure after radical prostatectomy for prostatic cancer without distant metastasis at a PSA of approximately 2.0 ng/mL is desirable; and it is thought to be important to continue LHRHa administration sufficiently until the level of PSA decreases to 0.01–0.02 ng/mL.

Analysis of patient characteristics in the two groups showed a significant difference in GS when radical prostatectomy was performed (Table 1). Based on this analysis, outcomes were significantly better in the IAD group (Figs 4 and 5). These results show that GS influenced the relapse rate in the CAD group while it did not influence the relapse rate in the IAD group. Leval et al compared the 3-year relapse rate between patients with GS ≤ 6 and those with GS ≥ 7 by examining prostate biopsies, and reported that the recurrence rate tended to be increased when GS increased in the CAD group (GS ≤ 6, 25.8%; GS ≥ 7, 71.4%) while there was no significant difference in the recurrence rate in the IAD group (GS ≤ 6, 6.2%; GS ≥ 7, 7.1%). In addition, they reported that the non-recurrence rate in patients with GS ≥ 7 was significantly lower in the IAD group than in the CAD group [20]. One possible explanation for this finding is that the growth of male hormone-independent tumors may occur more frequently with CAD in patients with a high GS. It is suggested that intermittent administration of LHRHa might be more effective than continuous administration in patients with high GS.

This study has several limitations. First, this was a retrospective study, and therefore information on the decision-making process for selecting the IAD and CAD groups is not available. In addition, the duration of LHRHa treatment is different between these groups. Second, there was a difference in the pathological background between the IAD and CAD groups. As a result, the favorable therapeutic outcome may also arise from the favorable pathology of the IAD patients compared with the CAD patients at the starting point. Third, there was no clear indicator for resumption and discontinuation of LHRHa administration and physicians’ judgment played a role in the timing of resumption and discontinuation. Fourth, we defined the initiation of oral anti-androgenic agent administration as recurrence in this study, and therefore our results could not be directly compared with those of other reports. Finally, the performance of a large-scale prospective study with a larger number of patients will be required to determine the optimal IAD protocol.

## Conclusions

Our retrospective study indicates that IAD therapy showed better oncological outcomes compared to CAD therapy. IAD therapy for PSA recurrence after radical prostatectomy is one of the effective treatment options from the viewpoint of reducing medical cost as well as non-recurrence rate, and improves the overall survival rate.
